# Saiga antelope horn suppresses febrile seizures in rats by regulating neurotransmitters and the arachidonic acid pathway

**DOI:** 10.1186/s13020-024-00949-3

**Published:** 2024-06-03

**Authors:** Wenxing Wu, Wencong Song, Jingjing Zhao, Sheng Guo, Min Hong, Jie Zheng, Yongqing Hua, Peng Cao, Rui Liu, Jin-ao Duan

**Affiliations:** 1 National and Local Collaborative Engineering Center of Chinese Medicinal Resources Industrialization and Formulae Innovative Medicine, Jiangsu Collaborative Innovation Center of Chinese Medicinal Resources Industrialization, and Jiangsu Key Laboratory for High Technology Research of Traditional Chinese Medicine Formulae, Nanjing University of Chinese Medicine, No 138 Xianlin Road, Nanjing, 210023 China; 2 School of Pharmacy, Nanjing University of Chinese Medicine, Nanjing, 210023 China; 3 Animal-Derived Chinese Medicine and Functional Peptides International Collaboration Joint Laboratory, Nanjing, 210023 China

**Keywords:** Saiga antelope horn Febrile seizures Metabolomics Network pharmacology

## Abstract

**Background:**

Saiga antelope horn (SAH) is a traditional Chinese medicine for treating febrile seizure (FS) with precise efficacy, but its mechanism of action and functional substances are still unclear. Given the need for further research on SAH, our group conducted studies to elucidate its mechanisms and active substances.

**Methods:**

An FS rat pup model was constructed through intraperitoneal injection of LPS and hyperthermia induction. Behavioural indicators of seizures, hippocampal histopathological alterations, serum levels of inflammatory cytokines and hippocampal levels of neurotransmitters were observed and measured to investigate the effects of SAH on FS model rats. Hippocampal metabolomics and network pharmacology analyses were conducted to reveal the differential metabolites, key peptides and pathways involved in the suppression of FS by SAH.

**Results:**

SAH suppressed FS, decreased the inflammatory response and regulated the Glu-GABA balance. Metabolomic analysis revealed 13 biomarkers of FS, of which SAH improved the levels of 8 differential metabolites. Combined with network pharmacology, a “biomarker-core target-key peptide” network was constructed. The peptides of SAH, such as YGQL and LTGGF, could exert therapeutic effects via the arachidonic acid pathway. Molecular docking and ELISA results indicated that functional peptides of SAH could bind to PTGS2 target, inhibiting the generation of AA and its metabolites in hippocampal samples.

**Conclusion:**

In summary, the functional peptides contained in SAH are the main material basis for the treatment of FS, potentially acting through neurotransmitter regulation and the arachidonic acid pathway.

**Graphical abstract:**

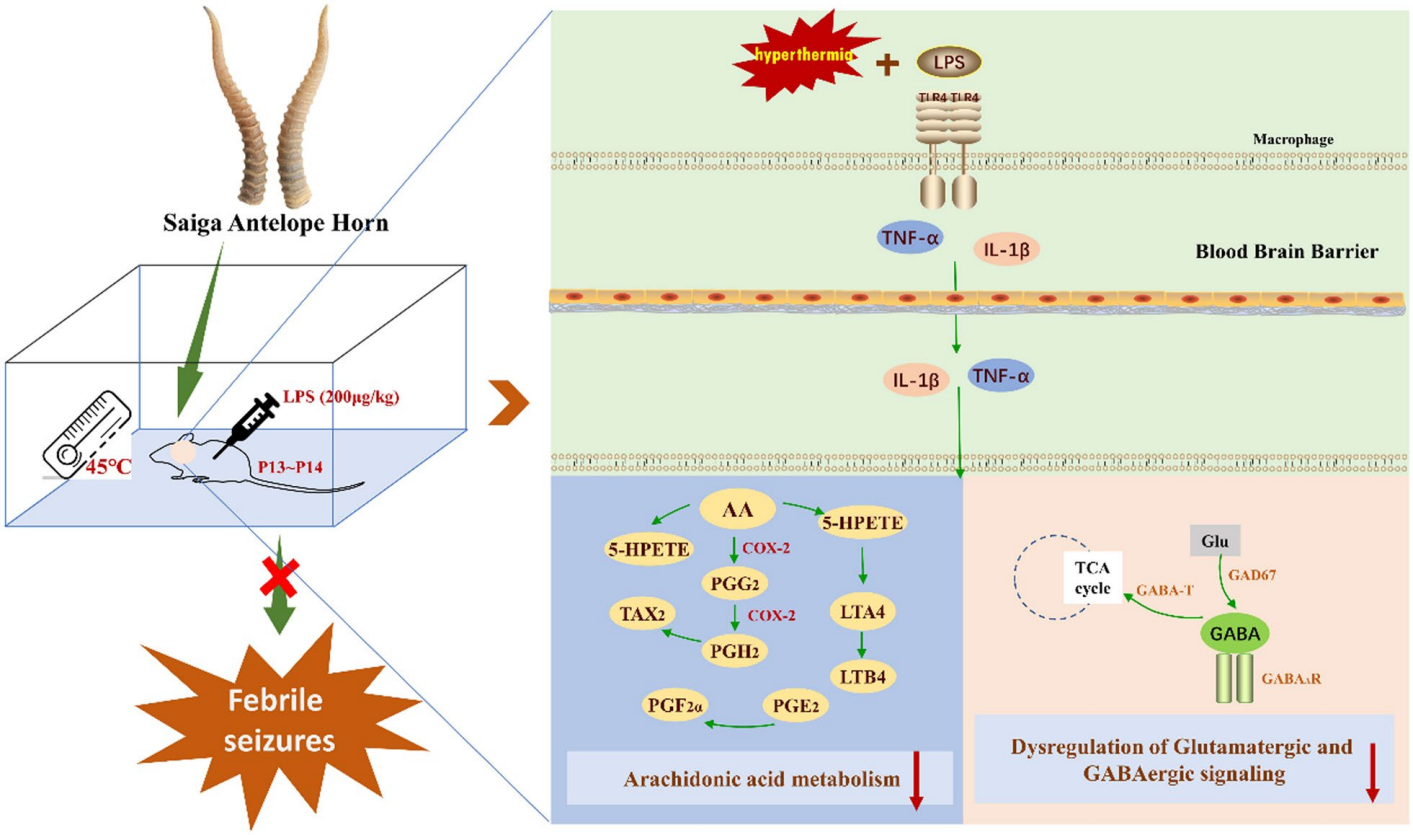

**Supplementary Information:**

The online version contains supplementary material available at 10.1186/s13020-024-00949-3.

## Introduction

Febrile seizure (FS) is a common paediatric problem [1]. The International League Against Epilepsy (ILAE) defines an FS as “a seizure in children over one month old, associated with fever but not with central nervous system infection, lacking prior neonatal seizures or unprovoked seizures, and not fitting other acute symptomatic seizure criteria”[2]. FS typically peaks at 18 months and occurs most frequently in children aged 6 months to 6 years [3]. Approximately 2–5% of children in Europe and the USA experience at least one fever-related convulsion before reaching five years of age [4, 5]. The prevalence is higher in certain Asian countries, reaching 8% in Japan and 16% in southern China [6]. Although most febrile seizures (FSs) seem benign, one-third last longer or recur within 24 h; such cases are complex and linked to an increased risk of developing temporal lobe epilepsy (TLE) later on [7, 8].

Current therapies for FS involve antipyretics such as paracetamol and ibuprofen [9, 10] and anticonvulsants such as diazepam and clobazam to prevent seizure recurrence [11, 12]. However, the high doses of anticonvulsants required can cause drowsiness and ataxia [13], potentially hindering the detection of serious illnesses by patients and doctors. Traditional Chinese medicine (TCM) categorizes FS as described by *Ji Jing Feng*. Clinical studies have shown that TCM is effective in preventing convulsive seizures and recurrence and has the same effect as Western medicine, with the advantages of few side effects and superior efficacy.

Saiga antelope horn (SAH) is the horn of Saiga antelope (*Saiga tatarica* Linnaeus), a precious animal derived from Chinese medicine. Its first recorded use appears in the “Divine Husbandman’s Classic of the Materia Medica” with a medicinal history spanning over 2000 years. SAH has the ability to restrain wind, relieve spasms, and clear and calm the liver and is widely used clinically for FS in children. Its Chinese patented drug, Antelope Horn Granules, is a representative prescription for the treatment of FS. It was found that Antelope Horn Granules have anticonvulsive effects and can inhibit the central nervous system and increase body resistance. Animal experiments have shown that SAH can decrease the body temperature of yeast-hyperthermia rats significantly [14] and inhibit the voluntary activity of mice. Consequently, the clinical efficacy of SAH treatment on FS has been clinically confirmed.

However, since the Saiga antelope is listed as a threatened species on the IUCN Red List [15], the clinical use of SAH is severely restricted. Therefore, it is urgent to find and evaluate alternatives to the SAH. However, the mechanism and functional material of SAH for treating FS are still unclear. The research on anticonvulsant functional material and mechanism of SAH is helpful to screen alternative resources with similar substance composition and similar mechanism of action with the aim of conserving Saiga antelope populations [16]. In this study, we developed a rat model for febrile seizures (FS) through combined hyperthermia and LPS injection. We measured convulsive behaviour, including convulsion occurrence, latency, and mortality rate, to comprehensively evaluate the anticonvulsive effect of SAH. We also investigated SAH’s effects on hippocampal pathological changes, and the levels of neurotransmitters and inflammatory factors in rat pups. Subsequently, potentially effective peptides and therapeutic mechanisms were screened and investigated via an integrative approach involving network pharmacology and hippocampal metabolomics.

## Materials and methods

### Materials

Saiga antelope horn (SAH) was obtained from Beijing Tongrentang Co., Ltd. and verified by Professor Jin-ao Duan at Nanjing University of Chinese Medicine. The horns were ground to a fine powder of less than 125 μm. SAH was prepared as a suspension in 0.2% CMC-Na for use in animal experiments. SAH analysis was conducted using Nano/LC‒MS/MS on a Q Exactive Plus quadrupole-Orbitrap mass spectrometer paired with a Dionex Ultimate 3000 nano-LC system (Thermo Fisher Scientific, San Jose, CA) [17]. The raw data obtained were imported into PEAKS Studio software (8.5 Edition) for analysis against the Saiga Keratin database. Detailed methodologies are provided in the supplementary materials.

### Establishment of FS model and drug treatment

Rat pups came from timed-pregnant Sprague–Dawley rats acquired from Beijing Vital River Laboratory Animal Technology, China (License SCXK (Jing) 2019-0010). The mother rats were kept in standard conditions with a temperature of 22 ± 2°C, relative humidity of 60 ± 2%, and 12 h light/dark cycles, with unlimited access to food and water. The birth time of the pups was recorded every 12 h, and the birth day was designated as postnatal day 0. Animal welfare and experimental procedures strictly followed the NIH Guide for the Care and Use of Laboratory Animals. The experiment was reviewed and approved by the Animal Experiment Ethics Committee of Nanjing University of Chinese Medicine (Approval No. 202208A002).

**Fig. 1:**
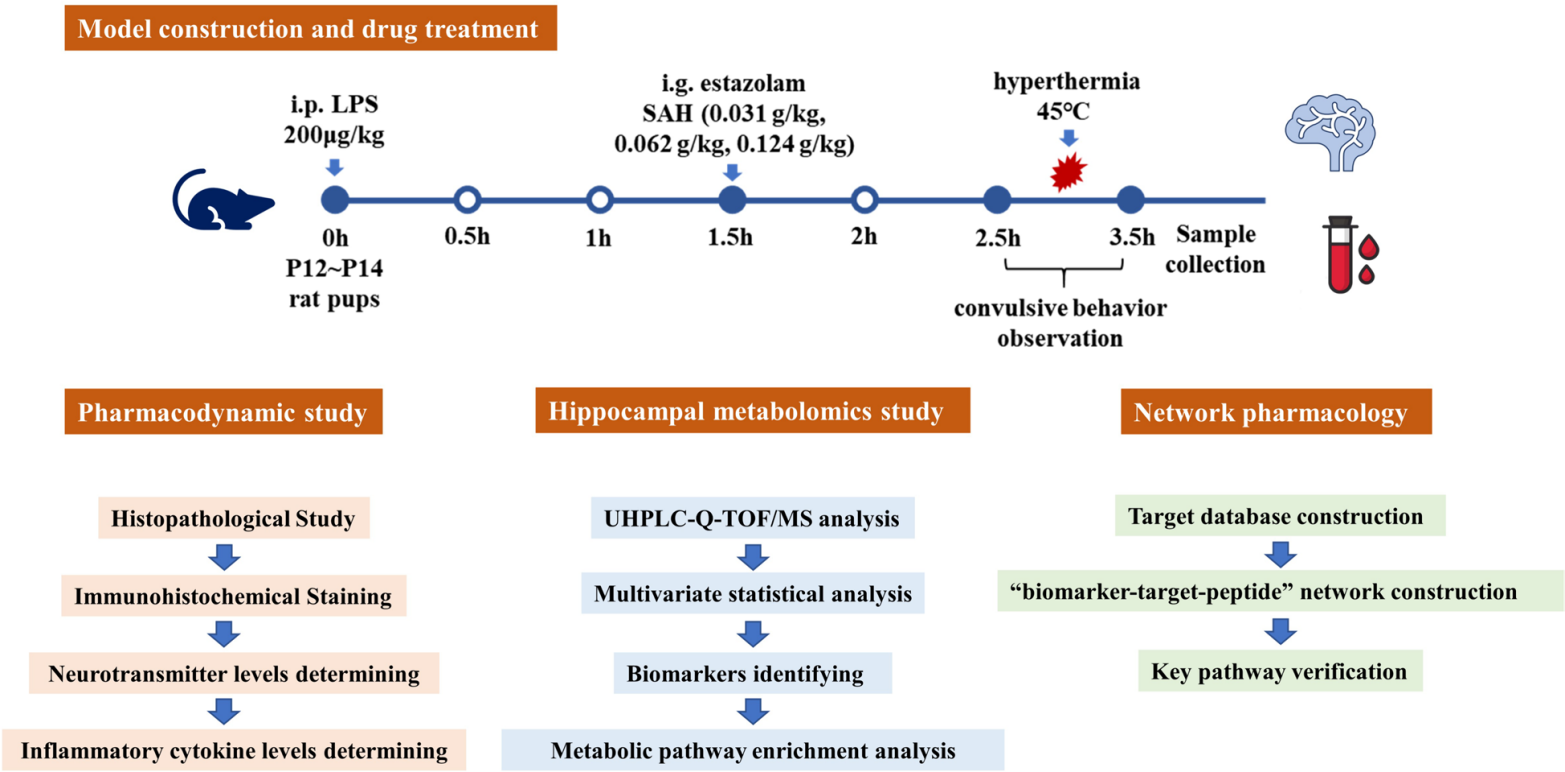
Overall design of experiments

A total of 120 rat pups (P12 ~ P14, male and female, ca. 50% each) were used and assigned into six groups randomly (n = 20): (1) control, (2) FS model, (3) Estazolam, (4) SAH-0.031 g/kg, (5) SAH-0.062 g/kg, and (6) SAH-0.124 g/kg. An FS rat model was constructed according to a protocol described previously [18]. Rat pups were separated from their dams and held in an incubator (Shanghai Yuyan Scientific Instrument Co.) at 30℃ for 30 min before seizure induction [19]. In addition to the control group, rat pups in other groups received LPS injection (*Escherichia coli* endotoxin 055: B5; Sigma Chemicals Co., St. Louis, MO; batch number: L2880, 200 μg/kg, i.p.) 2.5 h before hyperthermia induction to mimic fever (Fig. 1). Rat pups of control group were injected with equal volumes of sterile saline. All rat pups in each group were given the corresponding drug by gavage 1 h prior to hyperthermia induction. The rats in the Estazolam group were administered an estazolam suspension (0.62 mg/kg, Changzhou Siyao Pharmaceutical Co., Ltd., batch number: 20220813, 1 mg/tablet). Rats in the SAH-0.031 g/kg, SAH-0.062 g/kg, and SAH-0.124 g/kg groups were administered SAH (0.031, 0.062, 0.124 g/kg), respectively, while rats in the control group and FS group were treated with 0.2% CMC-Na (Sinopharm Chemical Reagent Co., Ltd., batch number: 20191203). Then rat pups were placed in the incubator at 45℃ to induce hyperthermic seizures for 30 min. The control group rats were maintained at 30℃. All rats were individually observed for seizure behaviour during hyperthermia period using the five-stage scale [20]:

- Stage 1 involves hyperactivity, twitching of the whiskers, and chewing.
- Stage 2 is characterized by head nodding, head clonus, and myoclonic jerks.
- Stage 3 features unilateral forelimb clonus.
- Stage 4 involves rearing with bilateral forelimb clonus.
- Stage 5 presents as a generalized tonic–clonic seizure (GTCS) with loss of the righting reflex.

An observer, unaware of the animal’s group assignments, conducted the seizure scoring. The time from hyperthermia induction to the first seizure occurrence, known as seizure latency, was measured and recorded. After behavioural assessment, the rats were euthanized using 1% sodium pentobarbital (50 mg/kg, i.p.). Blood and brain tissue samples were collected. Blood samples were centrifuged at 860 × g for 15 min at 4℃ to separate the serum. Whole brain tissues from three rats in each group were immersed and fixed in 4% paraformaldehyde (Biosharp, batch number: BL1515A) for later sectioning and pathological staining.

### Histopathological study and immunohistochemical staining

The fixed brain tissues were prepared into sections after paraffin embedding, dewaxing and hydration. Staining was performed according to the instructions of H&E staining kit (Servicebio Technology Co., Ltd., batch number: G1076) and Nissl staining kit (Servicebio Technology Co., Ltd., batch number: G1036). The sections were visualized under 200 × and 400 × high magnification, respectively. ImageJ software measured the number of neurons (N) and the area of the corresponding visual field (A) in three selected fields of view per section. The neuronal density was calculated as N/A.

Immunohistochemical (IHC) staining was used to detect the NeuN expression in hippocampal tissue. Brain tissue sections were deparaffinized and rehydrated to repair the antigen, incubated with 3% H_2_O_2_ for 15 min and rinsed with PBS buffer (Aifang Biotechnology Co., Ltd., batch number: AFIHC017). Then, 5% BSA (Servicebio Technology Co., Ltd., batch number: GC305010) was used for occlusion, followed by the addition of primary antibody (Anti-NeuN, Aifang Biotechnology Co., Ltd., batch number: AF300513, 1: 500) incubated at 4℃ overnight. On the following day, the secondary antibody (Aifang Biotechnology Co., Ltd., batch number: AFIHC001) was added after a full immersion in PBS buffer and incubated at room temperature for 30 min with PBS buffer. DAB (Aifang Biotechnology Co., Ltd., batch number: AFIHC004) was used to develop the color for 1 min, and the hematoxylin staining solution (Aifang Biotechnology Co., Ltd., batch number: AFIHC005) was used to re-stain for 3 min. Finally, the slices were dehydrated, transparent, and then sealed. The sections were visualized under 400 × high magnification. ImageJ software was used to determine the integrated option density (IOD) and the area of the positive region for each image. The mean density and percentage of positive area were calculated to indicate the relative expression of NeuN.

### ELISA and RT‒qPCR analysis

The serum frozen at −80 ℃ was thawed on ice, and the levels of tumor necrosis factor-α (TNF-α, batch number: AF3056-A), interleukin-1β (IL-1β, batch number: AF2923-A), S-100β (batch number: AF40339-A), and neuron-specific enolase (NSE, batch number: AF2945-A) in serum were measured using ELISA kits from Aifang Biotechnology Co., Ltd., following the manufacturer’s instructions. Hippocampal tissue (20 mg) was extracted with 180 μL of PBS buffer (Beyotime Biotechnology Co., Ltd., batch number: ST477). After resting at 4 ℃ for 30 min, the mixture was vortexed and centrifuged at 1000 × g for 15 min. The supernatant was collected to determined the levels of γ-aminobutyric acid (GABA, batch number: AF3317-A) and glutamic acid (Glu, batch number: AF3476-A) in the hippocampus according to the instructions of the ELISA kits from Aifang Biotechnology Co., Ltd. Protein concentrations in hippocampal supernatants were measured using a BCA protein assay kit (Beyotime Biotechnology Co., Ltd., batch number: P0010).

For RT-qPCR analysis, we extracted total RNA from brain tissues using TRIzol and reverse transcribed 4.2 μg of RNA using random primers and the EasyScript All-in-One First-Strand cDNA Synthesis SuperMix for qPCR (One-Step gDNA Removal, Transgene, Beijing, China). Concentrations and purities of RNA and cDNA were measured with a DS-11 spectrophotometer (DeNovix, Wilmington, DE, USA). RT-qPCR was conducted using an ABI 7500 real-time PCR system (Applied Biosystems, Waltham, MA, USA). Relative gene expression was calculated using GAPDH as a reference, following the 2^−∆∆Ct^ method. All primers were synthesized by Sangon Biotechnology (Table S1).

### Hippocampal metabolomics study

Hippocampal samples (50 mg) were extracted with 400 μL of 80% acetonitrile. After resting at 4 ℃ for 30 min, the mixture was vortexed and centrifuged at 16,000 × g for 15 min. The supernatant was transferred to liquid vials for intubation, and 2 μL was injected into a UHPLC-Q-TOF/MS for metabolic analysis. Chromatographic and mass spectrometry conditions can be found in the supplementary materials.

Data were imported into SIMCA P14.1 and scaled using Pareto scaling for multivariate statistical analysis, including principal component analysis (PCA) and orthogonal partial least squares-discriminant analysis (OPLS-DA). Scatter points in the OPLS-DA S-plot and variables with VIP > 1 were identified as potential biomarkers for further analysis [21]. T-tests were performed to determine if there were significant differences in the relative peak areas of biomarkers between groups, selecting metabolites with statistical significance (*P* < 0.05). The Human Metabolome Database (HMDB, http://www.hmdb.ca/) was utilized to identify potential markers [22]. Finally, differential metabolites were analysed for metabolic pathway enrichment using MetaboAnalyst 5.0 (http://www.metaboanalyst.ca/) [23].

## Integrated network pharmacology and metabolomics analysis

### Construction of the target database

Peptides identified from SAH based on Nano/LC–MS/MS analysis were subjected to ExPASy Peptide Cutter online software to obtain potential peptides digested with pepsin (pH 1.3) and trypsin. The Swiss Target Prediction database (http://www.swisstargetprediction.ch/) was employed to identify targets of digested peptides and hippocampal metabolomics biomarkers, using a probability > 0 filter and limiting to “*Homo sapiens*” [24]. The OMIM (https://www.omim.org/), Disgenet (https://www.disgenet.org/search) and GeneCards (https://www.genecards.org/) databases were searched with the keywords “Febrile convulsion” and “Febrile seizures” to gather targets related to FS. The targets of peptides, FS and metabolomics biomarkers are provided in the supplementary materials.

### Construction of the “biomarker-target-peptide” network

Peptides, biomarkers, and FS targets were uploaded to Venny 2.1 to obtain the intersecting targets, which may be potential targets for SAH to suppress febrile convulsions and improve hippocampal metabolic disorders. The “biomarker-target-peptide” network was conducted by importing the intersecting targets into Cytoscape (3.7.0) [25]. The Network Analyser tool analysed the network’s topological properties, identifying targets with above-average degree, betweenness centrality (BC), and closeness centrality (CC) as SAH’s core targets. The top 10 peptides, based on degree value, were identified as the key peptides for the efficacy of SAH. A “biomarker-core target-key peptide” network was constructed based on the analysis. Core targets were imported into the STRING database (https://cn.string-db.org/) to create a protein–protein interaction (PPI) map, assessing target interactions.

### Verification experiments

ELISA and molecular docking analyses were conducted to confirm the key targets and involved signalling pathways. All kits were provided by Aifang Biotechnology Co., Ltd. The 3D structures of key active peptides of SAH with the lowest energy conformations were generated in ChemBio3D Ultra 14.0.0.117 and saved in *mol2 files format. Hub target proteins’ 3D structures were sourced from the RCSB PDB database (https://www.pdbus.org/), with water molecules removed and hydrogens added to the proteins. All the target proteins and peptides were converted to *pdbqt files in AutoDock Vina software (https://vina.scripps.edu/) [26], and peptides were docked to the active sites of target proteins with the setting of spacing = 1.

### Statistical analysis

Results were presented as the mean ± standard error of the mean (SEM). Statistical significance was evaluated utilizing SPSS 22.0 software (IBM, Armonk, USA). Prior to selecting appropriate statistical tests, normal distribution of data (Shapiro–Wilk test) and homogeneity of variances (Levene’s test for equal variances) were checked. Data of incidence of convulsive behaviour and mortality rates were analysed using the chi-square test. Other normally distributed data were assessed using one-way analysis of variance (ANOVA) with LSD’s multiple comparisons test. Kruskal–Wallis test was used for non-normally distributed data analysis. *P* < 0.05 was considered to be a significant difference.

## Results

### Nano/LC‒MS/MS analysis of SAH

A total of 48 keratin and (unique peptides > 2) and 4018 peptides were identified from SAH based on the Nano/LC‒MS/MS analysis and keratin database of Saiga Antelope. According to the coverage and −10log*P values*, the top 10 keratins and peptides are listed in Tables 1 and 2. The mass spectrometry proteomics data have been deposited to the ProteomeXchange Consortium (https://proteomecentral.proteomexchange.org) via the iProX partner repository [27, 28] with the dataset identifier PXD052162.

**Table 1:** Keratins of SAH identified by the Saiga database (top 10 of −10lg*P*)

No.	Keratin	−10lg*P*	Coverage (%)	Peptides	Unique	Avg. mass	Description
1	SAH_KP8	521.87	98	348	177	55041	Keratin type II cuticular Hb6
2	SAH_KP9	509.81	99	331	136	48703	Keratin type II microfibrillar component 5
3	SAH_KP16	502.48	99	319	101	51538	Keratin type II microfibrillar component 7C
4	SAH_KP35	484.55	99	160	108	35474	Keratin type I cuticular Ha6
5	SAH_KP45	479.1	93	218	143	30640	Keratin type I microfibrillar 48 KD component 8C-1
6	SAH_KP57	472.4	91	96	96	14074	Keratin high-sulfur matrix protein IIIA3
7	SAH_KP72	449.38	99	154	53	21204	Keratin type II cuticular Hb1
8	SAH_KP34	436.22	98	91	65	11263	Keratin type I microfibrillar 47.6 KD
9	SAH_KP17	415.04	91	99	56	50228	Keratin type I cytoskeletal 42
10	SAH_KP50	399.96	75	102	64	53401	Keratin type II cytoskeletal 6A

**Table 2:** Peptides of SAH identified by the Saiga database (top 10 of −10lg*P*)

No*.*	Peptide (N–C)	−10lgP	Mass	Length	Keratin
1	RDVEAWFNTQTEELNQQVVSSSEQLQCCQTEIIELR	128.32	4367.044	36	SAH_KP35
2	RPVCCDPCSLQEGCCRPITCCPTSCQAVVCR	119.72	3842.579	31	SAH_KP57
3	NFSSCSLGGHLNYSGSSCGSSFPSNLVYSADLCPR	119.6	3783.635	35	SAH_KP13
4	GLLDSEDCKLPCNPCATTNACERPIGPCISNPCVSR	117.25	4117.821	36	SAH_KP34
5	TFYEAELAQMQTHISDTSVVLSMDNNR	117.18	3099.428	27	SAH_KP50
6	YSSQLAQMQGLIGNVEAQLAEIR	113.78	2534.28	23	SAH_KP35
7	TGSCCGPTFSSLSCGGGCLQPCGYRDPCCCRPVSCQTTVSRPVTSVPR	113.19	5426.3	48	SAH_KP57
8	SLRDHLHYSGSSCGSSFPSNLVYR	112.61	2725.267	24	SAH_KP29
9	MTGSCCGPTFSSLSCGGGCLQPCGYR	112.24	2856.122	26	SAH_KP57
10	YSSQLAQMQGLIGNVEAQLAEIR	110.61	2518.285	23	SAH_KP35

### SAH effectively prevented the onset of FS

**Fig. 2:**
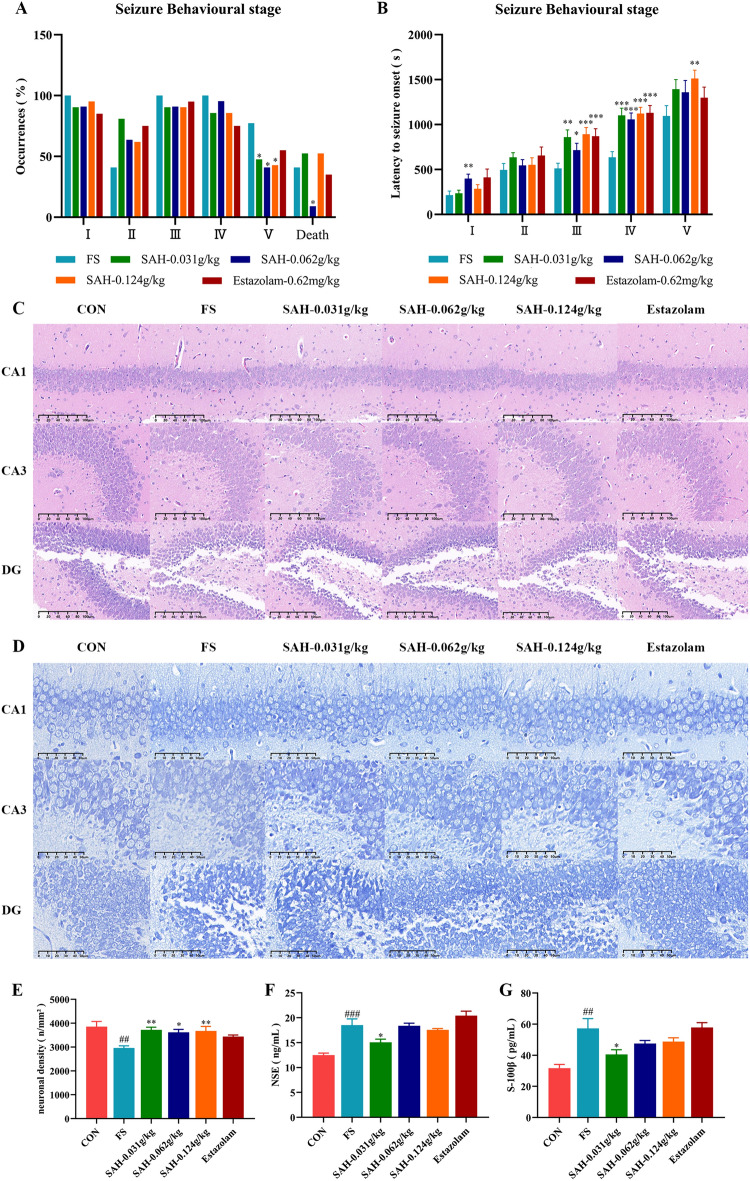
Effect of SAH on FS-induced seizure behaviour and histological damage to the hippocampus in rats. **A** The occurrence of seizures in the different groups (n = 20). **B** The latency to seizure onset in the different groups (n = 20). **C** Representative micrographs of the hippocampus with HE staining (200 × , n = 3). **D** Representative micrographs of the hippocampus with Nissl staining (400 × , n = 3). **E** Neuronal density in the hippocampus of rats (n = 3). **F** The concentrations of NSE and S100-β in the serum of the rats (n = 8). **G** The concentrations of NSE and S100-β in the serum of the rats (n = 8). The data in the figure are expressed as the mean ± SEM. ^*^P < 0.05 and ^**^P < 0.01 indicate differences from the FS group; ^##^*P* < 0.01 and ^###^*P* < 0.001 indicate differences from the control group

After LPS injection combined with incubation at 45 ℃, FS was successfully induced in the model group, aligning with prior findings that hyperthermia can effectively trigger seizures [18]. Rat pups of FS group displayed severe seizure-like symptoms, evolving into tonic–clonic convulsions. The incidence of convulsive behaviour at different stages and the seizure latency are shown in Fig. 2A, B. Compared to the control group, rats treated with SAH and estazolam experienced fewer seizures and had longer seizure latency. In addition, SAH treatment at 0.062 g/kg significantly decreased the occurrence of tonic‒clonic convulsions (*P* < 0.05) and the death rate of the rat pups (*P* < 0.05). Overall, SAH treatment effectively suppressed FS by delaying seizure onset, extending latency periods, and improving survival rates.

### SAH mitigated the histological damage in the rat hippocampus caused by FS

FS group exhibited significant changes in the number and morphology of hippocampal neurons compared to the control group after hyperthermia induction. In the control group, hippocampal neurons were tightly and uniformly arranged with distinct nuclei, whereas in FS group, neurons were shrunken with indistinct boundaries between the nucleus and cytoplasm (Fig. 2C). Hippocampal neurons of rats in SAH group showed fewer pathological changes compared to those in the FS group, being more abundant and closely arranged, with normal morphology, distinct nuclear-cytoplasmic boundaries, and prominent nucleoli. Additionally, the notable decrease in hippocampal neuronal density and increase in serum NSE and S100-β levels further indicated significant pathological damage in the hippocampal tissues of FS rats (Fig. 2E, F, G). In contrast, SAH treatment improved neuronal density and decreased the serum levels of NSE and S100-β. Accordingly, our experimental results showed that SAH relieved FS-induced histological damage to hippocampal neurons.

### SAH enhanced NeuN protein expression in the rat hippocampus

**Fig. 3:**
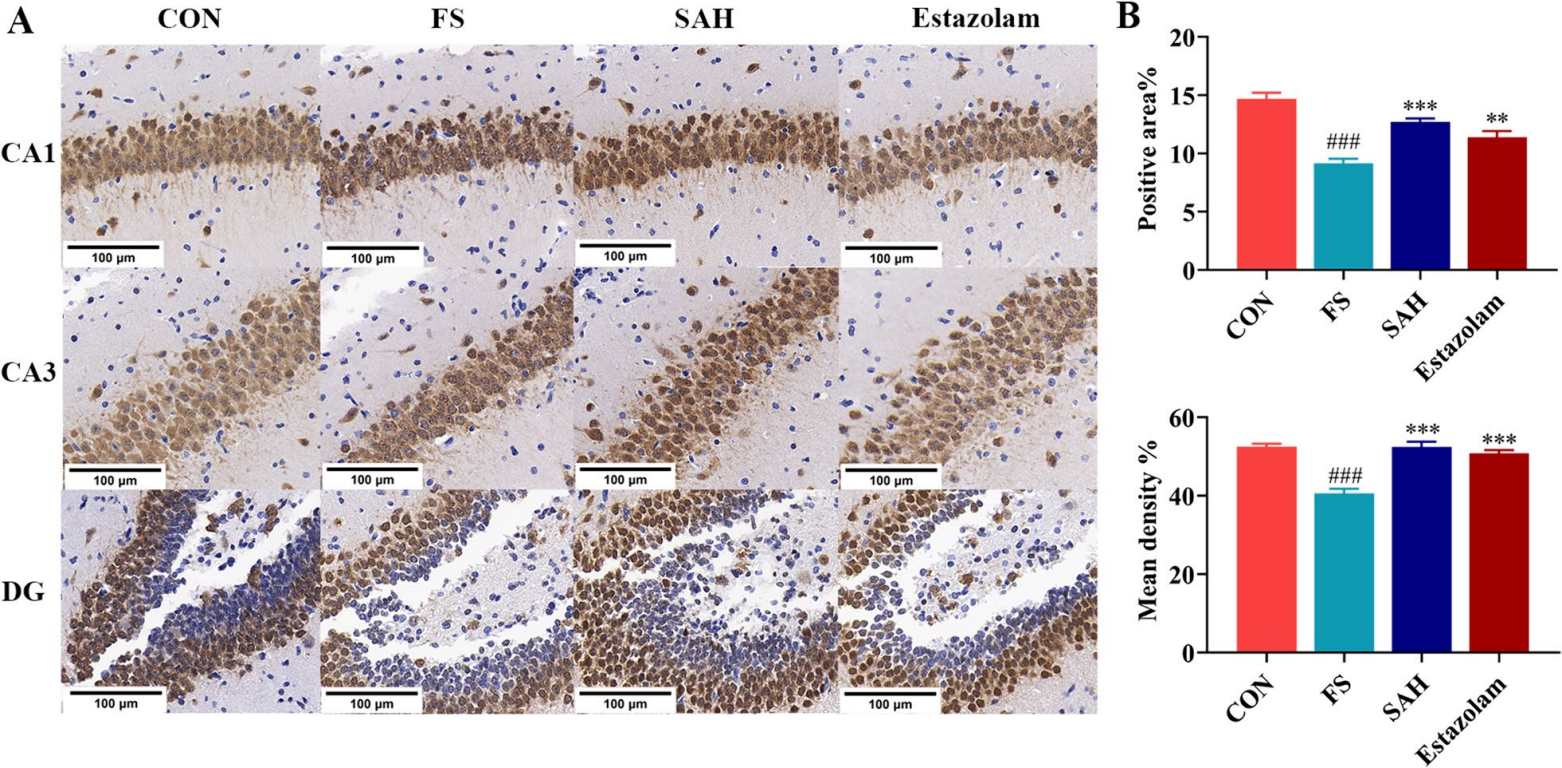
Effect of SAH on NeuN protein expression in the hippocampus of rats. **A** Representative micrographs of the hippocampus with IHC staining (400 ×). **B** The mean density and percentage of NeuN-positive cells. The data in the figure are expressed as the mean ± SEM, n = 3. ^**^P < 0.01, ^***^P < 0.001 represents a difference from the FS group; ^###^*P* < 0.001 represents a difference from the control group

NeuN is a mature neuronal marker that is usually used for the assessment of neuronal death or loss. As shown in Fig. 3, NeuN protein expression in the hippocampal tissues of FS group rat pups was significantly lower than in the control group (*P* < 0.001). However, SAH treatment enhanced NeuN protein expression (*P* < 0.001), suggesting that SAH powder can improve neuronal damage and exert neuroprotective effects in FS rats.

### SAH regulated neurotransmitter expression in FS rats

**Fig. 4:**
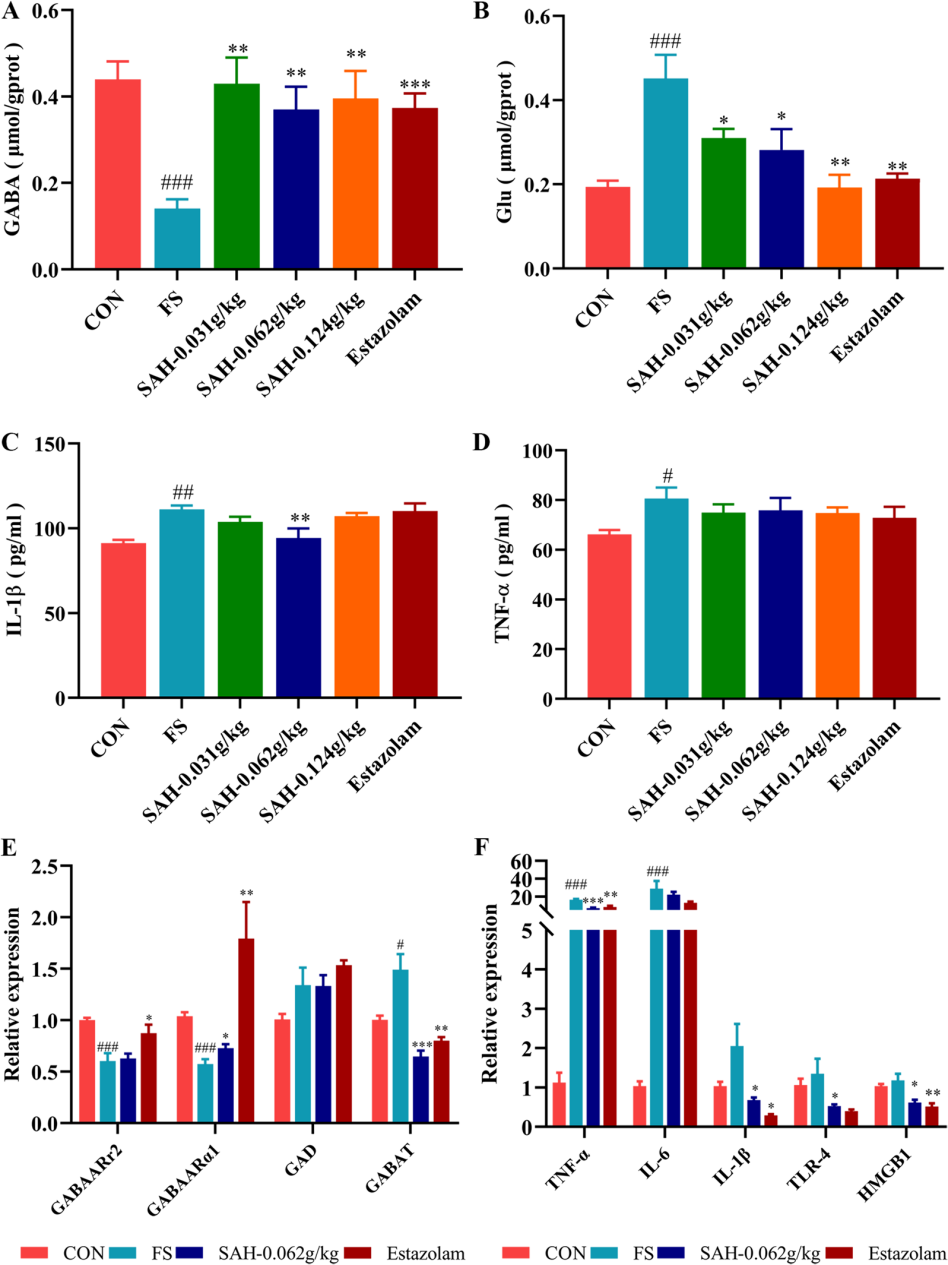
Effect of SAH on neurotransmitter and inflammatory cytokine levels in FS rats. **A** The levels of GABA in the hippocampus of the different groups (n = 8). **B** The levels of Glu in the hippocampus of the different groups (n = 8). **C** The levels of IL-1β in the serum of the different groups (n = 8). **D** The levels of TNF-α in the serum of the different groups (n = 6). **E** The mRNA levels of receptors related to GABA in the hippocampus of the different groups (n = 6). **F** The mRNA levels of inflammatory cytokines and their receptors in the hippocampus of the different groups (n = 6). The data in the figure are expressed as the mean ± SEM. ^*^P < 0.05, ^**^P < 0.01, and ^***^P < 0.001 represent differences from the FS group; ^#^*P* < 0.05, ^##^*P* < 0.01, and ^###^*P* < 0.001 represent differences from the control group

We further explored the regulation of neurotransmitters in the hippocampus of rats with FS-induced SAH. As shown in Fig. 4A, B, the levels of GABA (*P* < 0.001) in the hippocampus of FS group rats were markedly decreased, and the levels of Glu in the hippocampus were markedly increased compared to the control group (*P* < 0.001). Furthermore, FS reduced the mRNA levels of GABAARα1 and GABAARγ2 while increasing GABAT mRNA levels (Fig. 4E). These results indicated that LPS combined with hyperthermia contributes to the disruption of hippocampal neurotransmitter levels in rats, which subsequently leads to abnormal neuronal discharges. Compared with those in the FS group, the GABA, GABAARα1 and GABAARγ2 levels were clearly increased, and the Glu and GABAT levels were significantly reduced in the SAH group. Overall, SAH treatment protected neurons by regulating the levels of neurotransmitters and their receptors, reducing the excitability of the nervous system and causing damage to neuronal cells.

### SAH inhibited the increase in levels of inflammatory cytokine in FS rats

To assess SAH’s effect on inflammation after FS, serum levels of IL-1β and TNF-α were measured. Serum concentrations of IL-1β and TNF-α were significantly higher in the FS group compared to the control group but were reduced in the SAH and estazolam group compared to the FS group (Fig. 4C, D). The SAH-0.062 g/kg group exhibited a significant decrease in IL-1β levels (*P* < 0.01). Additionally, inflammatory factors mRNA levels and their receptors in hippocampal tissues were determined by qPCR. After hyperthermia induction, mRNA levels of IL-1β, IL-6, TNF-α, TLR4 and HMGB1 in the FS group were higher than those in the control group. SAH administration significantly reduced the levels of IL-6, TNF-α, TLR4, and HMGB1 in the SAH-0.062 g/kg group (Fig. 4F). These results indicate that SAH effectively inhibits the release of inflammatory factors in vivo, suppress hippocampal neuroinflammation, and alleviate brain damage in rat pups.

### SAH regulates abnormal changes in hippocampal metabolic profiles in FS rats

**Fig. 5:**
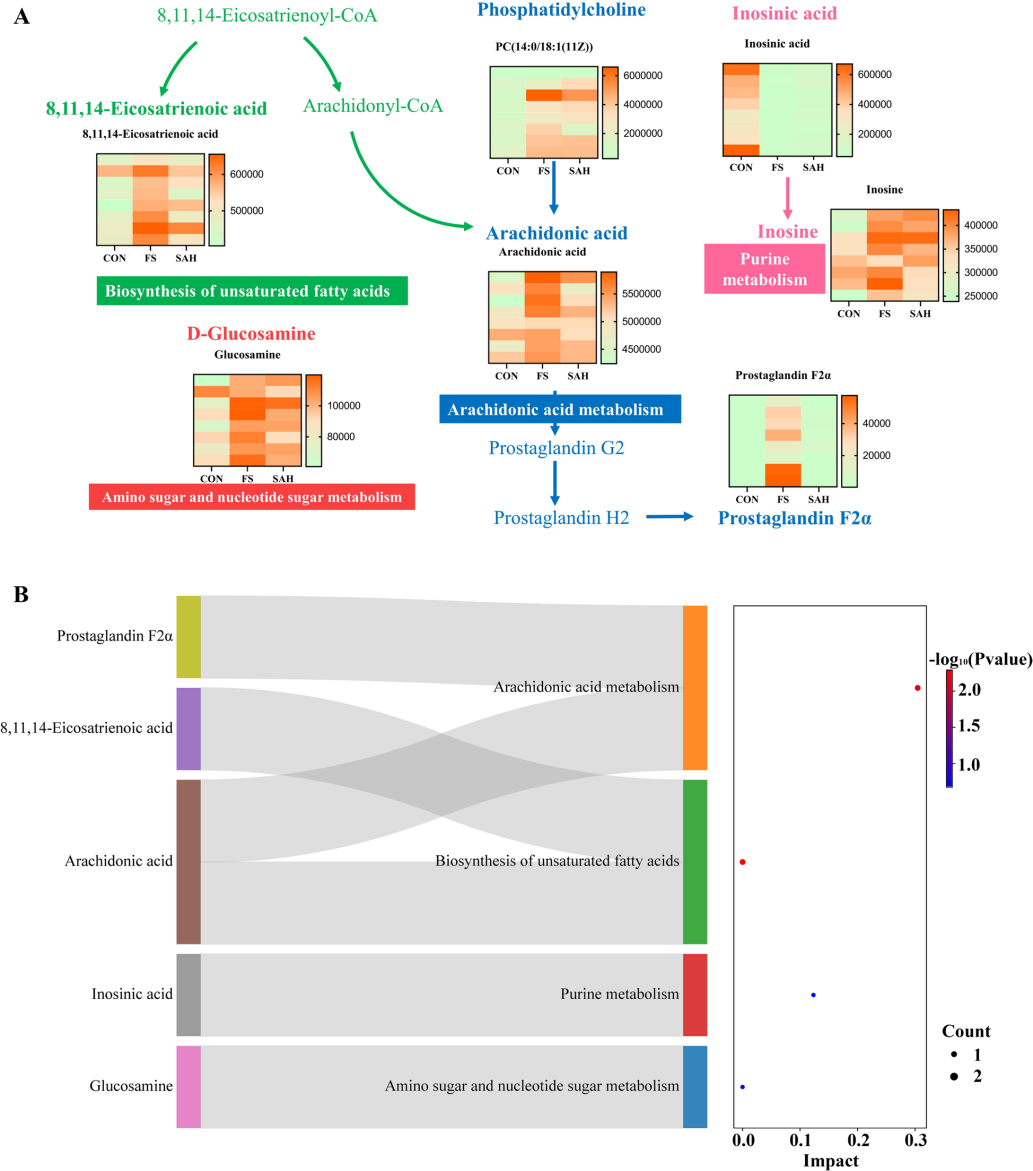
Effect of SAH on metabolic pathways in the hippocampus of FS rats (n = 8). **A** The network of metabolomic features involved in the protection of SAH against FS via multiple pathways. **B** Metabolic pathways of anticonvulsive action in SAH

UHPLC-QTOF/MS offers a fast, efficient, and convenient approach to analyse chemical differences between various rat samples. This method was utilized to gather metabolic information from the hippocampus in both positive and negative ion modes. The metabolic raw data have been deposited to the MetaboLights [29] (http://www.ebi.ac.uk/metabolights/login) with identifier MTBLS10140. PCA and OPLS-DA analyses of the chromatographic data were conducted using SIMCA-P, a multivariate statistical analysis software. The PCA score plot showed significant clustering of different groups in both ion modes, highlighting substantial metabolic differences between the SAH, control, and FS groups (Fig. S1). The OPLS-DA score plot displayed obvious separation between the FS and control groups in both ion modes (Fig. S1). This indicates significant changes in the metabolic profiles of rats in the FS group compared to the control group, attributable to FS. Subsequently, potential markers of interest were identified from the S-plots constructed after OPLS-DA. Using MS/MS data, KEGG, and HMDB 3.6, 13 metabolites in the hippocampal samples were identified and annotated. Mass spectrometry data and the trends for the FS group versus the control group are detailed in Table S2. The differential ion concentrations in the hippocampus likely indicate changes in endogenous metabolites due to FS. The levels of 8 biomarkers, including prostaglandin F2α and arachidonic acid, were reversed after the administration of SAH (Table S2 and Fig. 5A). These results indicated that SAH treatment can effectively modulate the abnormalities in these potential biomarkers. To identify pathways affected by SAH, 8 biomarkers were analysed using the MetaboAnalyst database and R studio software to delineate metabolic pathways. Figure 5B demonstrates that SAH can alter the arachidonic acid metabolic pathway and the unsaturated fatty acid biosynthesis pathway.

### Network pharmacology results of the SAH treatment of FS

**Fig. 6:**
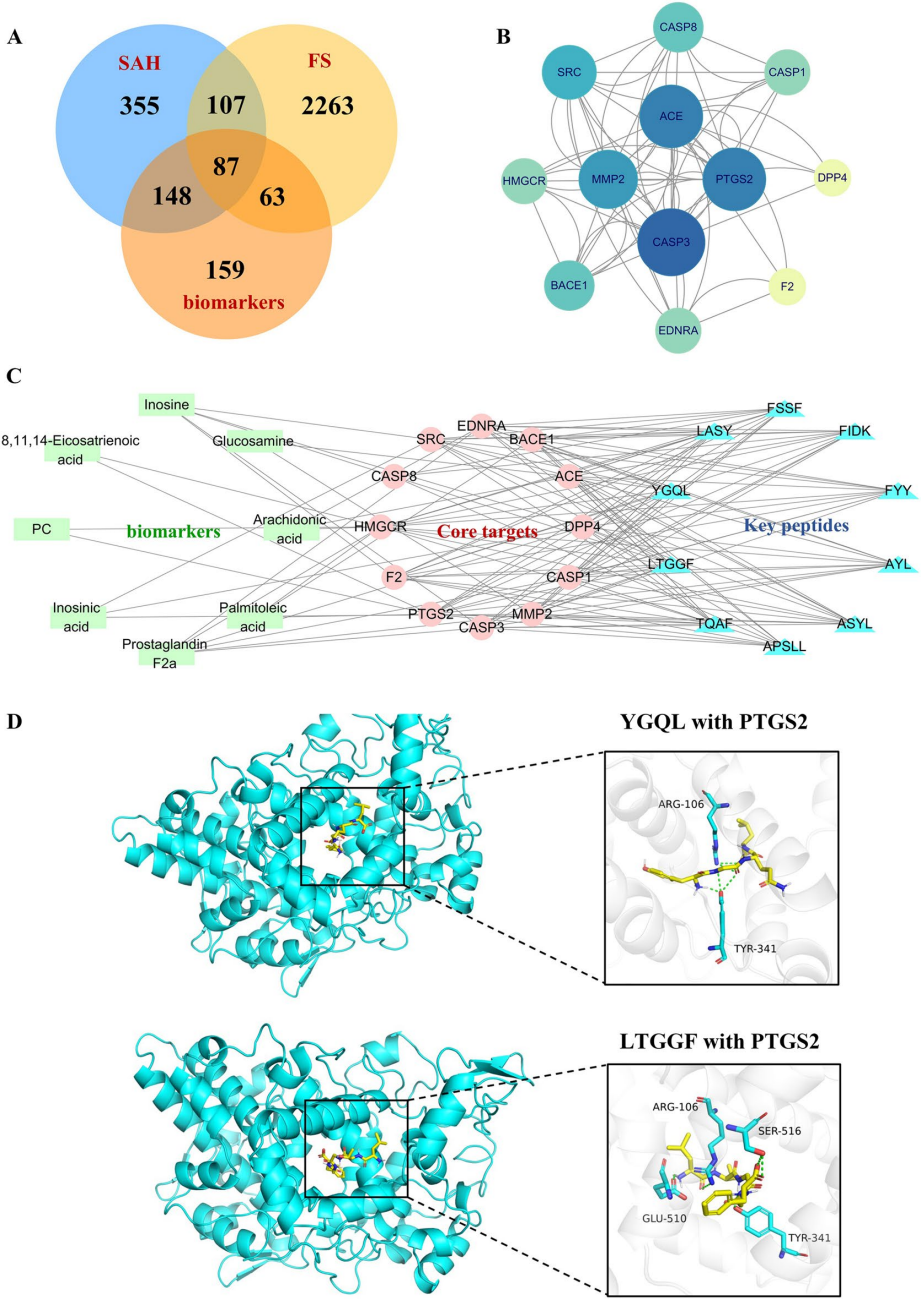
Network pharmacology analysis of the treatment of FS with SAH. **A** Distribution of SAH, FS, and biomarker target genes; **B** Protein‒protein interaction network of 12 core targets of SAH for treating FS; **C** “Biomarker-Core Target-Key Peptide” network; **D** Molecular docking results

In the present work, a total of 2244 peptides containing 2  ~  20 amino acids were obtained after virtual digestion. A total of 697 targets of 2244 peptides from SAH and 457 targets of biomarkers were predicted based on Swiss Target Prediction. A total of 2520 targets related to FS were collected from OMIM, Disgenet and GeneCards databases. After removing false positives and duplicates, 87 intersecting targets were identified through Venny software for further investigation (Fig. 6A), which may be potential targets for the efficacy of SAH. Then, the network of “biomarkers-targets-peptides” was constructed and its topological properties were analysed with the function “Network analyse” in Cytoscape 3.7.0 software. Degree, BC and CC are important parameters for measuring the criticality of a node in the network. Based on the topological properties, we found 12 targets with values greater than average (Table S3), which exert a hub role and may be core targets for SAH to suppress febrile seizures and regulate hippocampal metabolic disorders. In addition, the top 10 peptides were ranked according to their degree and regarded as the key peptides for the efficacy of SAH treatment. The association information of “biomarker-core target” and “key peptide-core target” were used to construct the network of “biomarker-core target-key peptide” (Fig. 6C) through Cytoscape 3.7.0 software. The PPI network of the core targets was constructed using the String database, which contains 12 nodes and shows the interaction relationships of target genes (Fig. 6B).

### SAH suppresses the arachidonic acid metabolic pathway to treat FS

Metabolomics and network pharmacology analyses revealed that the arachidonic acid metabolic pathway as the crucial pathway involved in the effects of SAH on FS. The ten core peptides and the target PTGS2 (COX2), associated with the arachidonic acid metabolic pathway, were selected for molecular docking analysis. Generally, the lower the energy required to stabilize the ligand-receptor binding conformation, the higher the likelihood of interaction. This study used a binding energy of ≤ −5.0 kcal/mol as the screening criterion. The molecular docking results, presented in Table 3, indicate that the binding energies of the 10 key anticonvulsant peptides in SAH with PTGS2 were all below −5 kcal/mol (except for APSLL). The peptides with the lowest binding energies were YGQL (−7.5 kcal/mol) and LTGGF (−7 kcal/mol), which were derived from SAH_KP58 (Keratin, high-sulfur matrix protein, B2A) and SAH_KP16 (Keratin, type II microfibrillar, component 7C), respectively. Consequently, the active peptide of SAH could combine with PTGS2 to form a stable conformation with high binding activity (Fig. 6D).

**Table 3:** Summary of the molecular docking binding energies of the core peptides and PTGS2 (unit: kcal/mol)

No.	Peptides	PTGS2	Keratin
1	APSLL	−4.1	Keratin, type I cytoskeletal 42
2	ASYL	−6.1	Keratin, type I cytoskeletal 42
3	AYL	−6.2	Keratin, type I cytoskeletal 42
4	FYY	−6.9	Keratin-associated protein 13-1
5	FIDK	−6.7	Keratin, type II cuticular Hb6
6	FSSF	−6.2	Keratin-associated protein 19-3
7	YGQL	−6.8	Keratin, high-sulfur matrix protein, B2A
8	LASY	−7.0	Keratin, type II microfibrillar, component 7C
9	LTGGF	−6.9	Keratin, type II cuticular Hb1
10	TQAF	−7.5	Keratin, high-sulfur matrix protein, B2A

**Fig. 7:**
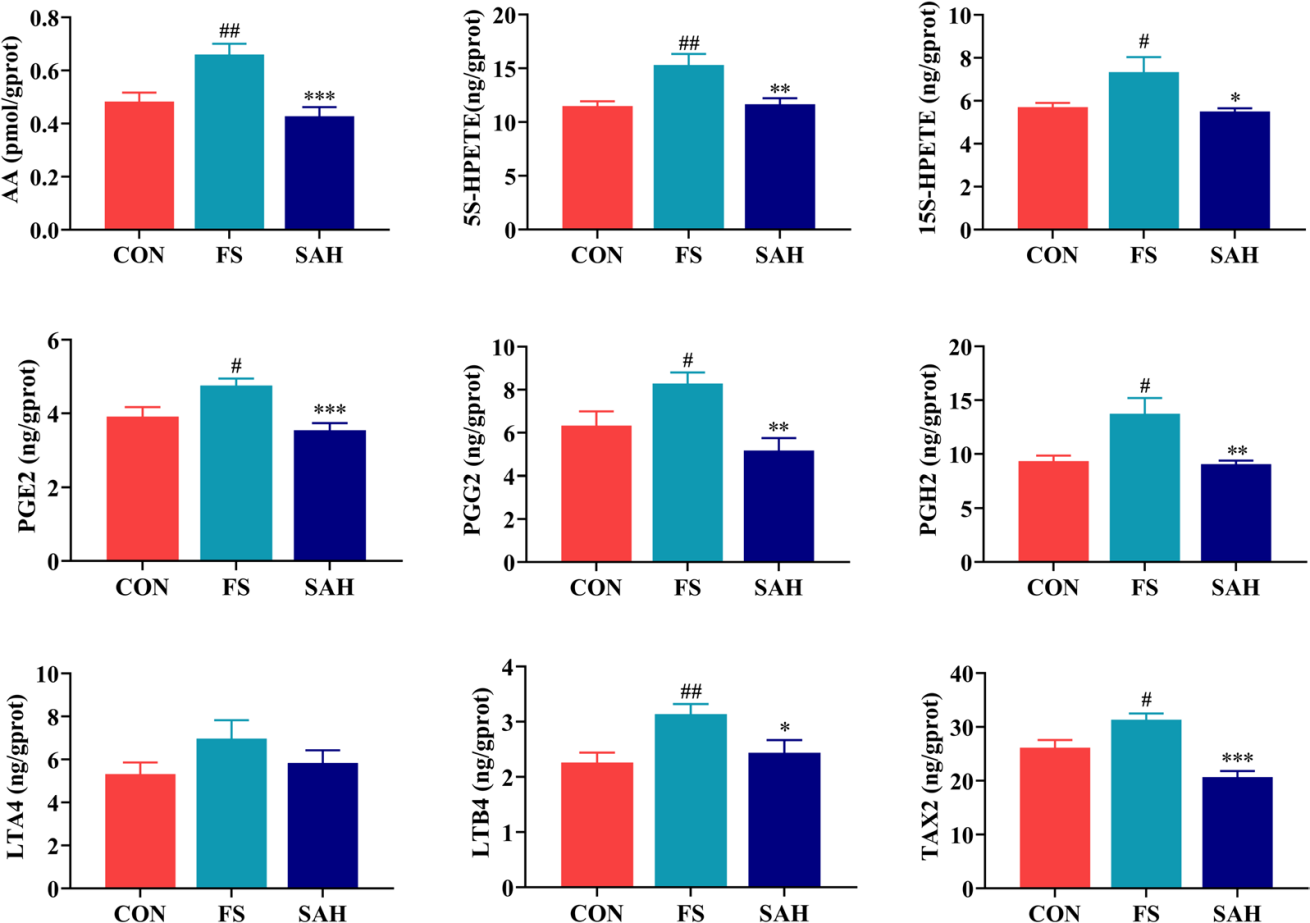
The levels of metabolites involved in the arachidonic acid metabolic pathway in the hippocampus of the different groups. The data in the figure are expressed as the mean ± SEM, n = 8. ^*^P < 0.05, ^**^P < 0.01, and ^***^P < 0.001 represent differences from the FS group; ^#^*P* < 0.05, ^##^*P* < 0.01, and ^###^*P* < 0.001 represent differences from the control group

To further validate the involvement of the arachidonic acid metabolic pathway, ELISA was used to measure levels of prostaglandin E2 (PGE2), prostaglandin G2 (PGG2), prostaglandin H2 (PGH2), leukotrienes A4 (LTA4), leukotrienes B4 (LTB4), arachidonic acid (AA), 5(S)-HPETE, 15(S)-HPETE, and thromboxane A2 (TAX2) in hippocampal tissues. Figure 7 shows that serum levels of AA, LTB4, PGE2, PGH2, PGG2, 5(S)-HPETE, 15(S)-HPETE, and TAX2 were significantly higher in the FS group. Conversely, the SAH group showed significantly lower levels of these metabolites compared to the FS group. These findings suggest that SAH may inhibit the production of AA and its metabolites, as illustrated in Fig. 7. Regulating the arachidonic acid metabolic pathway might be a key mechanism by which SAH treats FS.

## Discussion

FS is among the most frequent pediatric emergencies, highly prevalent in children under 6. Post-treatment, 30–40% of these children may experience recurrence [30]. Prolonged convulsive seizures and untimely and repeated seizures can cause a lack of oxygen in the brain, which can result in brain damage, affecting a child’s intelligence or leading to diseases such as epilepsy. FS is characterized by neuronal hyperexcitability due to an increase in core body temperature during fever, stemming from an underlying systemic infection [31, 32]. A variety of bacterial, viral, and other pathogenic infections are also common triggers of FS. The immune system is relatively weakened in children, and the excessive inflammatory response caused by infections can lead to excitotoxicity, which promotes convulsive seizures [33]. In addition, Genetic factors might also contribute to FS, with children who have a family history of FS being more prone to its development [34]. Current research has found that the pathogenesis of FS is mainly related to factors such as neurotransmitter imbalance, neuroinflammatory response and abnormal ion channel function caused by elevated body temperature [35]. The metabolic rate of the brain increases in a feverish state, leading to increased release of excitatory neurotransmitters such as glutamate, which increases neuronal excitability. In addition, elevated body temperature may also affect the function of GABA receptors, further heightening nervous system excitability.

In this study, we used LPS intraperitoneal injection combined with hyperthermia induction to establish a rat pup model of FS. Rats were examined on postnatal days 12–14, a period during which rat brain development closely mirrors the stage at which human infants are most vulnerable to FS [36]. Administering LPS (200 μg/kg) increased body temperature in rat pups, reduced their seizure susceptibility and threshold temperature. The LPS combined hyperthermia model effectively simulates key features of infection-associated febrile seizures (FS), closely resembling the clinical scenario [18]. LPS, found in the cell walls of gram-negative bacteria, is widely used to induce fever and mimic infection across various species [19, 37]. Toll-like receptors (TLRs), particularly TLR4, recognize LPS components, triggering the activation of the innate immune response [38]. This process leads to the transcription of both pro-inflammatory and anti-inflammatory cytokines. The subsequent cytokine production and secretion by peripheral macrophages initiate a cascade of downstream cytokines.

Elevated levels of pro-inflammatory cytokines, including TNF-α and IL-1β, along with increased TLR4 receptor transcripts, were observed in both the hippocampus and serum of young rats with hyperthermic convulsions. This finding indicated that LPS activated the inflammatory response in the rat pups. IL-1β is known to significantly impact the generation of convulsions, particularly febrile seizures [39]. Furthermore, metabolomics analysis revealed that FS induced abnormalities in the arachidonic acid metabolic pathway in the rat hippocampus. In FS rats, arachidonic acid and its metabolites were significantly increased. IL-1β affects both excitatory (glutamatergic) and inhibitory (GABAergic) brain circuits. Glutamate serves as the primary excitatory amino acid neurotransmitter in the central nervous system (CNS) [40]. Glutamate has a strong excitotoxic effect on neurons [41], and high concentrations of Glu can activate glutamate receptors, leading to a large amount of Ca^2+^ influx and Ca^2+^ overload in neurons [42], which induces a series of calcium-promoted reactions, generating irreversible cellular damage and causing neuronal degeneration and necrosis. GABA significantly inhibits the excitotoxic effects of glutamate (Glu) [43]. Glu generates large amounts of GABA under the action of GAD. GABA could inhibit glutamate release as a negatively feedback and further reduce the brain damage caused by Glu. An increase in hippocampal glutamate levels with a decrease in GABA levels was observed in FS rats. These results also demonstrate the important role of neuroinflammatory response and neurotransmitter homeostasis in FS. At present, the treatment of FS usually involves direct modulation of the neurotransmitter system, for example, by increasing GABAergic transmission or inhibiting excessive release of Glu [11]. However, these therapeutic strategies are often accompanied by certain side effects such as somnolence, reduced attention span, and even cognitive dysfunction [44], and such adverse effects are especially pronounced in pediatric patients [12].

We observed that SAH effectively modulated GABA and Glu levels in the hippocampus, revealing the potential role of SAH in correcting the imbalance between excitatory and inhibitory neurotransmitters in FS model rats. Specifically, SAH treatment could restore neurotransmitter balance and reduce neurotoxicity due to excessive neuronal excitation. SAH also exhibits a modulatory effect on the inflammatory response, which is closely related to neuroinflammation, another key pathogenesis of FS. It was found that SAH treatment was able to significantly reduce the expression of inflammatory factors such as IL-1β and TNF-α in the hippocampus, indicating that SAH attenuated FS-induced neuroinflammatory responses through anti-inflammatory effects. This effect may be achieved through direct inhibition of inflammatory signalling pathways or modulation of metabolic pathways related to inflammation. As shown in this study, SAH was able to effectively modulate the arachidonic acid metabolic pathway, which is closely related to inflammation.

In addition, the topological properties of the “biomarker-target-peptide” network indicated that the key target of SAH in interfering with the occurrence of FS was PTGS2 (COX-2). It is well known that PTGS2 is the rate-limiting enzyme for prostaglandin synthesis [45] and plays a key role in the early inflammatory response to an insult, and consequently a significant role in postseizure inflammation and hyperexcitability of the brain. PTGS2 activation catalyzes the production of the prostaglandin PGE2, which enters the hypothalamic region to further cause fever. Studies have shown that PTGS2 mRNA expression is induced in major hippocampal neurons within hours of seizures onset, thus PTGS2 has an important role in seizures and is potential neurotherapeutic target for seizure treatment [46]. These results suggest that SAH can suppress FS through multiple pathways, which is a safer and more effective option in clinical practice. Further through a combination of network pharmacology and metabolomics analyses, key active peptides of SAH for the treatment of FS were identified. Among these peptides, YGQL and LTGGF were identified as the peptides with the highest scores. YGQL is composed of tyrosine, glycine, glutamine, and leucine. Tyrosine is a precursor to dopamine and increases the seizure threshold [47]. Glycine, glutamine, and leucine inhibit neural excitation, which can reduce the occurrence of convulsions [48, 49].

This study revealed that SAH employs a multi-pathway mechanism for the treatment of FS, offering a uniquely valuable and safer clinical treatment strategy. The therapeutic action characteristics of SAH for the treatment of FS contributes to the development of new therapeutic strategies for the treatment of FS in the clinic. This discovery also provides a scientific basis for the screening and evaluation of SAH-like efficacy resources. The key peptides and targets obtained in this study also provide a clear direction for the development of substitutes to SAH. By screening medicinal material with similar biological activities or other natural sources, new therapeutic drugs can be developed, which not only helps to protect the endangered species, but also meets the clinical needs.

## Conclusion

In conclusion, SAH demonstrated positive anti-convulsive effects in FS model rats. It effectively suppressed FS. Furthermore, SAH was found to reduce inflammatory responses and rebalance neurotransmitter levels. SAH significantly reduced hippocampal tissue damage caused by FS. Combined with network pharmacology analysis, our findings confirmed that SAH affects FS by regulating differential metabolite levels, including AA, LTB4, and PGE2, involved in arachidonic acid metabolism. Additionally, YGQL and LTGGF were identified as the key functional peptides of SAH. Given these findings, research on alternatives to SAH presents a broader scope for future research.

### Supplementary Information

Additional file 1 Supplemental Method 1. Nano-LC-MS/MS analysis of SAH. Supplemental Method 2. Chromatographic and mass spectrometry conditions. Table S1. Gene specific primer pairs used in RT-qPCR. Table S2. Identification and trends of change for potential biomarkers. Table S3. The topological properties of core targets. Fig. S1. PCA, OPLS-DA score plots and S-Plot score plot of hippocampus samples collected from the control group, FS group, and SAH group based on UPLC-Q-TOF/MS. 1: positive ion modes, 2: negative ion modes.

Additional file2 The collected targets of Saiga antelope horn, febrile seizures and biomarkers.

## Data Availability

The datasets used and/or analysed during the current study are available from the corresponding author on reasonable request.

## Abbreviations

FS: Febrile seizures
GABA: γ-Aminobutyric acid
Glu: Glutamate
IHC: Immunohistochemistry
IL-1β: Interleukin-1β
ILAE: International league against epilepsy
LPS: Lipopolysaccharide
NSE: Neuron-specific enolase
OPLS-DA: Orthogonal partial least-squares-discriminate analysis
PCA: Principal component analysis
SAH: Saiga antelope horn
SPF: Specific pathogen-free
TCM: Traditional Chinese medicine
TNF-α: Tumor necrosis factor-α
