# Supplementary material for: Saiga antelope horn suppresses febrile seizures in rats by regulating neurotransmitters and the arachidonic acid pathway
Source: Chin Med. 2024 Jun 3;19:78. doi: 10.1186/s13020-024-00949-3 (PMC11149251; doi:10.1186/s13020-024-00949-3)
Supplement: Supplementary file 1 — Additional file 1 Supplemental Method 1. Nano-LC-MS/MS analysis of SAH. Supplemental Method 2. Chromatographic and mass spectrometry conditions. Table S1. Gene specific primer pairs used in RT-qPCR. Table S2. Identification and trends of change for potential biomarkers. Table S3. The topological properties of core targets. Fig. S1. PCA, OPLS-DA score plots and S-Plot score plot of hippocampus samples collected from the control group, FS group, and SAH group based on UPLC-Q-TOF/MS. 1: positive ion modes, 2: negative ion modes. [file 13020_2024_949_MOESM1_ESM.pdf]

Saiga Antelope Horn Suppresses Febrile Seizures in Rats by Regulating Neurotransmitters and the Arachidonic Acid Pathway

Wenxing Wu [a, b, c], Wencong Song[a, b], Jingjing Zhao [a, b], Sheng Guo[a, b], Min Hong[b], Jie Zheng[b], Yongqing Hua[b], Peng Cao[a, c], Rui Liu[a, b, c*], Jin-ao Duan[a, b, c*]

a. National and Local Collaborative Engineering Center of Chinese Medicinal Resources Industrialization and Formulae Innovative Medicine，Jiangsu Collaborative Innovation Center of Chinese Medicinal Resources Industrialization，and Jiangsu Key Laboratory for High Technology Research of Traditional Chinese Medicine Formulae, Nanjing University of Chinese Medicine，Nanjing 210023, China.

[b] *School of Pharmacy, Nanjing University of Chinese Medicine, Nanjing 210023, China*

[c] *Animal-Derived Chinese Medicine and Functional Peptides International Collaboration Joint Laboratory, Nanjing 210023, China*

*Correspondence:

Rui Liu and Jin-ao Duan, Jiangsu Collaborative Innovation Center of Chinese Medicinal Resources Industrialization, Nanjing University of Chinese Medicine, No 138 Xianlin Road, Nanjing, 210023, China. E-mail: liurui@njucm.edu.cn (R. Liu) and dja@njucm.edu.cn (J.A. Duan), Tel: +86-25-85811524 (R. Liu) and +86-25-85811291 (J.A. Duan).

The following is the Supplementary data to this article:

Supplemental Method 1. Nano-LC-MS/MS analysis of SAH

1.1 Sample preparation

The proteins of SAH were extract as described with slight modifications. 10 mg of animal horn powder was immersed in 2 mL of 50 mM sodium phosphate (pH 7.8) with 2% sodium dodecyl sulfate (SDS) and 20 mM dithiothreitol (DTT), and then incubated overnight at 65°C. To the mixture, 20 μL of 100 mM iodoacetamide (IAA) was added to alkylate Cysteine residue at room temperature for 30 min in the dark. The proteins were precipitated with 5.5 times the volume of 80% acetone overnight, and then followed by 80% acetone wash. The pellet was resuspended in 200 μL of 50 mM Tris (pH = 8) containing 8 M urea. After sonicating in an ice-water bath for 20 min, the supernatant was achieved with centrifugation at 14,000 × g for 5 min. Then the supernatant was diluted with 50 mM Tris solution (pH = 8) to urea concentration below 1 M. Protein digestion was performed by adding trypsin at a 50:1 protein/enzyme ratio at 37°C for 16 h. Subsequently, 10% TFA was added until pH lower than 3 to quench the reaction. The digested peptide samples were desalted using SepPak C18 solid-phase extraction cartridge (Waters, Massachusetts, USA) and then dried in Vacuum Concentrator (Labconco, Kansas City, MO, USA). The peptides were reconstituted in 0.1% formic acid (FA) prior to MS analysis.

1.2 Nano-LC-MS/MS analysis

Samples were analyzed by LC-MS/MS on a Q Exactive Plus quadrupole-Orbitrap mass spectrometer (Thermo Fisher Scientific, San Jose, CA) coupled with a Dionex

Ultimate 3000 nanoLC system (Thermo Scientific). 2 μL of each sample was injected and separated on a lab-fabricated reverse phase capillary column (75μm × 15cm, particle size 1.7 μm, pore size 150 Å). A gradient of mobile phase A (0.1% FA in pure water) and B (0.1% FA in 98% acetonitrile) as 5%-20% B over 42 mins, 20%-25% B over 5 mins, 25%-35% B over 5 mins, 35%-80% B over 4 mins, and then holding 80% B for the last 4 mins was employed with a flow rate of 300 nL/min.

The instrument was operated in a data-dependent acquisition under positive mode to automatically switch between full-scan MS and MS/MS. Full-scan MS spectra were acquired with a threshold ion count of 10,000, an isolation width of 2.0 Da, and dynamic exclusion set to 30.0 second. The top 20 most abundant precursor ions were subsequently fragmented in orbitrap by higher energy collision-induced dissociation (HCD) with a target value of 50,000 ions. Source conditions were as follows: electrospray voltage of 2.0 kV and normalized collision energy set at 30. For Full-scan MS and MS/MS, the *m/z* scan range were set to 350-1800 and 200-2000, respectively.

1.3 Protein identification

The raw data were searched via PEAKS Studio Software (8.5 Edition, Bioinformatics Solutions Inc., Waterloo, Canada). The search was performed against the Saiga Keratin database. Search settings included the following: trypsin digestion with missed cleavages allowed to 2, MS1 tolerance of 10 ppm, MS2 tolerance of 0.02 Da, identified peptides filtered to curate a dataset with a false discovery rate (FDR) less than 1% at both the peptide and protein levels, static modification of 57.02 Da on cysteines representing carbamidomethylation from iodoacetamide treatment, dynamic modification of 15.99 Da on methionine representing oxidation, dynamic modification of 42.01 Da on N-terminal of a peptide representing acetylation, and dynamic modification of 0.98 Da

on Gln and Asn representing deamidation.

Supplemental Method 2. Chromatographic and mass spectrometry conditions

The separation was performed on an ACQUITY UPLC BEH C_{18} column (2.1 mm × 100 mm, 1.7 μm), which was maintained at 35 °C, and the mobile phase was composed of 0.1% formic acid solution (A) and acetonitrile (B) at a flow rate of 0.4 mL/min. The gradient elution conditions were as follows: 0.0-3.0 min, 5%–45% B; 3.0-13.0 min, 45%-95% B; 13.0-14.0 min, 95% B; 14.0-15.0 min, 95%-5% B. Electrospray ionization (ESI) mass spectra were acquired in both positive and negative ionization modes by scanning over the m/z range of 100-1000 Da. The conditions were as follows: extraction voltage, 2.0 V; cone voltage, 30 V; capillary voltage, 3.0 kV; collision energy, 20–50 eV; ion source temperature, 120 °C; desolvation temperature, 350 °C; cone gas flow rate, 50 L/h; and desolvation gas flow rate, 600 L/h. High purity nitrogen and leucine-enkephalin (ESI+: 556.2771 *m/z*, ESI−: 555.2615 *m/z*) were the gas collision and locked mass solution, respectively.

Supplemental table.

Table S1 Gene specific primer pairs used in RT-qPCR.

Gene	Sequence (5'-3')	
IL-1β	IL-1β-F	ATGCCTCGTGCTGTCTGACC
	IL-1β-R	TTTGTCGTTGCTTGTCTCTCCTTG
IL-6	IL-6-F	CTTCCAGCCAGTTGCCTTCTTG
	IL-6-R	TGGTCTGTTGTGGGTGGTATCC
TLR2	TLR2-F	AGACTCTGGAAGCAGGTGACAAC
	TLR2-R	GCAGGCGAGGCGGAGAATC
TLR4	TLR4-F	CTATCATCAGTGTATCGGTGGTCAG
	TLR4-R	ACAGCCAGCAATAAGTATCAGGTG
TNF-α	TNF-α-F	GAAACACACGAGACGCTGAA
	TNF-α-R	AGGGAGGCCTGAGACATCTT
HMGB1	HMGB1-F	CTAGCCCTGTCCTGGTGGTATT
	HMGB1-R	CCAATTTACAACCCCCAGACTGT
GAD	GAD-F	TGCTTCCAGCTAAGAACGGG
	GAD-R	CGATCAAACGTCTTGCGGAC
GABAT	GABAT-F	GTCCGAAGGTGGAGACAACC
	GABAT-R	CCAGTCTGAACCTCGTCCAC
GABAARα1	GABAARα1-F	TTGACTGTGAGAGCCGAATG
	GABAARα1-R	CAGAGCCGAGAACACGAAG
GABAARγ2	GABAARγ2-F	ACTATGTGGTTATGTCCGTGTA
	GABAARγ2-R	TGTGTATCCTCCCGTGTC
GAPDH	GAPDH-F	AGACAGCCGCATCTTCTTGT
	GAPDH-R	CTTGCCGTGGGTAGAGTCAT

Table S2 Identification and trends of change for potential biomarkers.

No.	t_R/min	Biomarker	ESI	*m/z*	Formula	Content variance	
						FS *vs* CON	SAH *vs* FS
1	0.79	Inosinic acid	-	347.0413	$C_{10}H_{13}N_4O_8P$	↓##	↑***
2	0.93	Inosine	-	267.0737	$C_{10}H_{12}N_4O_5$	↑##	↓
3	4.89	Prostaglandin F2α	-	353.2327	$C_{20}H_{34}O_5$	↑###	↓***

4	10.98	Docosahexaenoic acid	-	327.2334	$C_{22}H_{32}O_2$	↑	-
5	11.13	Palmitoleic acid	-	253.2175	$C_{16}H_{30}O_2$	↑##	↓*
6	11.21	Arachidonic acid	-	303.2334	$C_{20}H_{32}O_2$	↑##	↓**
7	11.21	Glucosamine	-	403.1581	$C_6H_{13}NO_5$	↑###	↓
8	11.63	8,11,14-Eicosatrienoic acid	-	305.2487	$C_{20}H_{34}O_2$	↑###	↓*
9	11.83	Adrenic acid	-	331.2643	$C_{22}H_{36}O_2$	↑	-
10	12.01	Palmitic acid	-	255.2331	$C_{16}H_{32}O_2$	↓##	-
11	8.83	LysoPC(18:1(9Z))	+	522.356	$C_{26}H_{52}NO_7P$	↑###	-
12	11.67	PC(14:0/18:1(11*Z*))	+	754.5389	$C_{40}H_{78}NO_8P$	↑##	↓
13	12.17	PC(14:0/16:0)	+	706.5383	$C_{38}H_{76}NO_8P$	↑#	-

↑: means increase; ↓: means decrease; -: means no significant difference. $^{*}P < 0.05$, $^{**}P < 0.01$, $^{***}P < 0.001$.

Table S3. The topological properties of core targets.

No.	**Core targets**	**BC**	**CC**	**Degree**
1	BACE1	0.3890	0.6338	634
2	ACE	0.2009	0.5749	558
3	F2	0.0812	0.4847	403
4	PTGS2	0.0602	0.4713	375
5	DPP4	0.0539	0.4608	352
6	CASP1	0.0474	0.4542	337
7	CASP8	0.0531	0.4449	315
8	MMP2	0.0195	0.4150	238
9	HMGCR	0.0698	0.4128	233
10	CASP3	0.0184	0.4114	228
11	SRC	0.0172	0.3829	143
12	EDNRA	0.0171	0.3775	124

Supplemental figure.

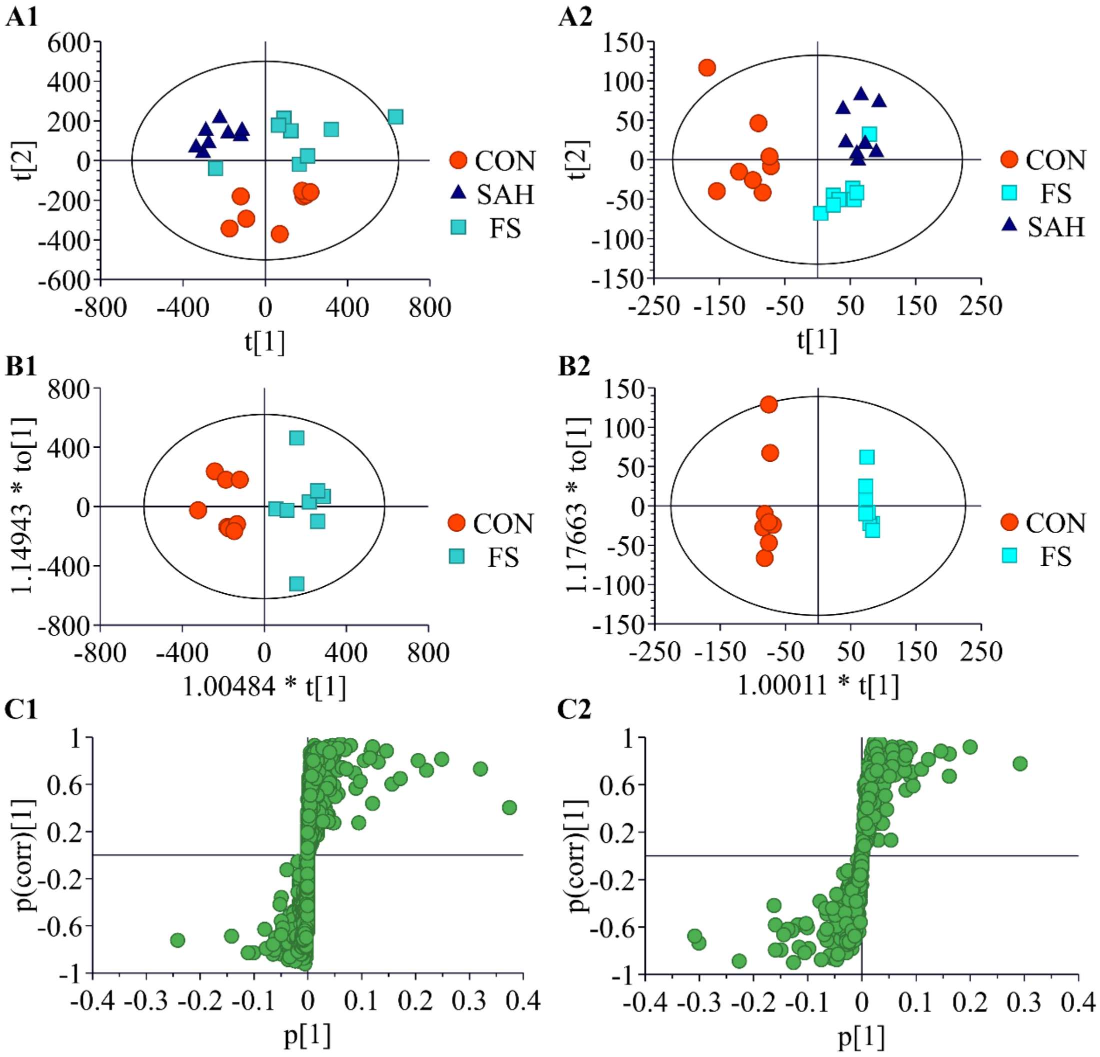

Fig. S1. PCA, OPLS-DA score plots and S-Plot score plot of hippocampus samples collected from the control group, FS group, and SAH group based on UPLC-Q-TOF/MS. 1: positive ion modes, 2: negative ion modes.
